# Unexpected air pollution with marked emission reductions during the COVID-19 outbreak in China

**DOI:** 10.1126/science.abb7431

**Published:** 2020-06-17

**Authors:** Tianhao Le, Yuan Wang, Lang Liu, Jiani Yang, Yuk L. Yung, Guohui Li, John H. Seinfeld

**Affiliations:** 1 Division of Geological and Planetary Sciences, California Institute of Technology, Pasadena, CA, USA.; 2 State Key Laboratory of Loess and Quaternary Geology, Institute of Earth Environment, Chinese Academy of Sciences, Xi’an, Shaanxi, China.; 3 Key Lab of Aerosol Chemistry and Physics, Institute of Earth Environment, Chinese Academy of Sciences, Xi’an, Shaanxi, China.; 4 Divisions of Chemistry and Chemical Engineering and Engineering and Applied Science, California Institute of Technology, Pasadena, CA, USA.

## Abstract

The absence of motor vehicle traffic and suspended manufacturing during the COVID-19 pandemic in China produced a unique experiment to assess the efficiency of air pollution mitigation. Up to 90% reduction of certain emissions during the city-lockdown period can be identified from satellite and ground-based observations. Unexpectedly, extreme particulate matter levels simultaneously occurred in northern China. Our synergistic observation analyses and model simulations show that anomalously high humidity promoted aerosol heterogeneous chemistry, along with stagnant airflow and uninterrupted emissions from power plants and petrochemical facilities, contributing to severe haze formation. Also, because of non-linear production chemistry and titration of ozone in winter, reduced nitrogen oxides resulted in ozone enhancement in urban areas, further increasing the atmospheric oxidizing capacity and facilitating secondary aerosol formation.

The abrupt outbreak of the Coronavirus Disease 2019 (COVID-19) pandemic produced unprecedented societal impacts in China. To curb the virus spread among humans, a preventive lockdown was first implemented on January 23 in Wuhan, Hubei. Other major cities/counties in China subsequently followed suit, and the entire nation’s lockdown lasted for at least three weeks (varying in different regions). During the lockdown period, emissions from the traffic sector were drastically reduced. Such a shutdown serves as a natural experiment to evaluate air quality responses to a dramatic emissions reduction and to assess the interplay between emission, atmospheric chemistry, and meteorological conditions. Here, we synthesize multiple-year satellite retrieved atmospheric compositions, national ground station measurements of major pollutants, meteorology from reanalysis data, and a suite of state-of-the-art online atmospheric chemistry model simulations to assess the atmospheric influence of the COVID-19 outbreak in China and to reveal its implications for air pollution control strategies.

China has continued to battle particulate haze pollution (*1*). Long-term regulatory plans targeting energy and industrial emissions have been implemented (*2*), and nation-wide improvement of fine particulate matter levels has been reported (*3*). Nonetheless, the key chemical and physical processes responsible for severe haze formation in China remain elusive, including exacerbated ozone levels (*4*, *5*), pathways of secondary aerosol formation (*6*, *7*), and emissions-meteorology interactions (*8*). Certain societal events in China with short-term stringent emission controls have been studied as natural experiments, such as the “Olympic Blue” during the 2008 Beijing Summer Olympic Games (*9*) and the “APEC Blue” during the 2014 Asia-Pacific Economic Cooperation (APEC) Economic Leaders’ Meetings in Beijing (*10*, *11*). Emission controls during these two events resulted in 40-60% reduction in SO_2_, NO_2_, non-methane volatile organic compounds (VOCs), and particulate matter.

The primary focus period during the COVID-19 lockdown in China was from January 23 to February 13, 2020 (hereafter referred to as the 2020-CLD period). This period encompassed a 7-day national holiday traditionally celebrating the Lunar New Year, during which previous studies have noted the reduction in anthropogenic emissions (*12*). Nitrogen dioxide (NO_2_) is key in atmospheric chemistry and serves as an important precursor for both ozone production and secondary aerosol formation (*6*, *13*). Changes in NO_2_ during the lockdown period can be assessed by comparing spaceborne NO_2_ measurements in the same time periods over different years. The TROPOspheric Monitoring Instrument (TROPOMI) on board the Copernicus Sentinel-5 Precursor satellite has provided key trace gas measurements of high accuracy since 2018. TROPOMI data show quite low column-integrated NO_2_ amount during the 2020-CLD, with a mean value of 1.72 mg m^−2^, and general uniformity throughout the whole country (Fig. 1A). By contrast, in the same period in 2019, hot spots of NO_2_ were evident over eastern China, where the regional mean NO_2_ abundance was 4-5 times higher than that in other regions of China (Fig. 1B). Regional means over eastern China experienced a 5.70 mg m^−2^ reduction in NO_2_, corresponding to a −71.9% fractional change (Fig. 1C). At the peak of the disease outbreak, Wuhan experienced a 93% fractional reduction in NO_2_. Such a short-term human-induced reduction in NO_2_ is unprecedented, well exceeding the previous 2014 “APEC-Blue” with largest NO_2_ reduction of about 40% (*10*). Compared to a five-year climatology (2015-2019) based on the NASA Aura Ozone Monitoring Instrument (OMI), the NO_2_ reductions mainly occurred over the North China Plain (fig. S1).

**Fig. 1:**
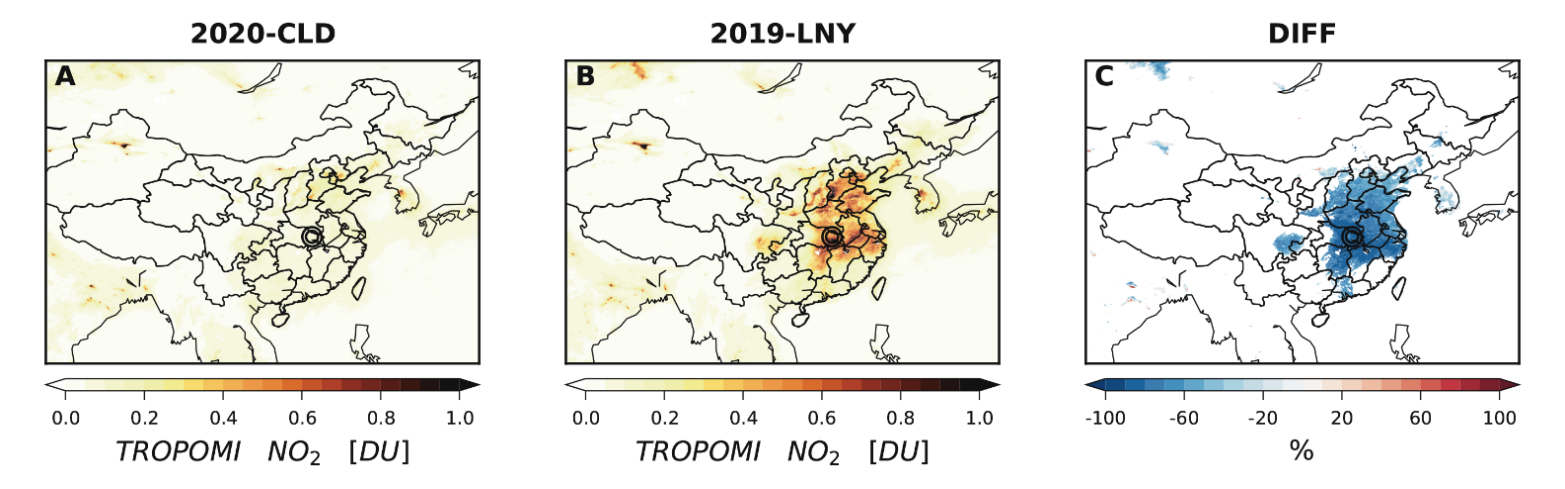
Spaceborne measurements of NO_2_ from TROPOMI. (**A**) Column-integrated NO_2_ averaged over the COVID19 lockdown period (CLD) for three weeks during Jan. 23 to Feb. 13, 2020. (**B**) Column-integrated NO_2_ averaged over the reference period in 2019. To account for the annual holiday, the 2019 reference period we choose is the same as that in 2020-CLD in the Chinese lunar calendar, starting from the two days before the Chinese Lunar New Year (2019-LNY). Note that TROPOMI NO_2_ is available only starting from June 2018. (**C**) The fractional changes between (A) and (B), calculated only for the regions with NO_2_ in 2019-LNY greater than 0.2 DU. The symbols in the maps indicate the location of Wuhan, the most affected city by the COVID-19 disease. 1 Dobson Unit (DU) = 0.4462 mmol m^−2^.

In addition to spaceborne retrievals, we explore surface measurements of fine-mode aerosols and trace gas species over entire eastern China (fig. S2). We calculate separately the climatological means of the past five years (2015-2019) during the same three-week period as the CLD, including the Lunar New Year (hereafter referred to as CLIM-LNY) and the same three-week period in the Georgian Calendar (CLIM). The difference between CLIM-LNY and CLIM is attributed mainly to the holiday effect. In Wuhan, surface concentrations of NO_2_ and SO_2_ were the lowest compared with the three-week means before the CLD as well as the climatological means over the past five years. PM_2.5_ (particulate matter with aerodynamic diameter less than 2.5 μm) was reduced by 23.2 μg m^−3^ (−32.4%) and 37.4 μg m^−3^ (−43.5%) as compared to CLIM-LNY and CLIM, respectively (Fig. 2A). In contrast to the changes to the PM_2.5_, surface ozone mixing ratio showed a +5.0 ppb (+25.1%) enhancement in Wuhan during the CLD as compared to CLIM-LNY. Ozone chemistry is highly nonlinear, and in the wintertime urban areas in China, its production is in a NO_x_-saturated regime (NO_x_ = NO + NO_2_) due to the relative lack of HO_x_ radicals (*13*). Besides, reduction of fresh NO emissions alleviates ozone titration (*13*, *14*). Thus, a reduction of NO_x_ leads to an increase in ozone. Previous studies also attributed the anticorrelation between PM_2.5_ and ozone to the aerosol radiative effect on the photochemistry of ozone formation (*4*, *15*) as well as the aerosol sink for ozone precursors (*5*). Changes in gaseous and particulate levels in the major cities of southern China, Guangzhou (Fig. 2C) and Shanghai (Fig. 2D), resemble those of Wuhan during the city lockdown.

**Fig. 2:**
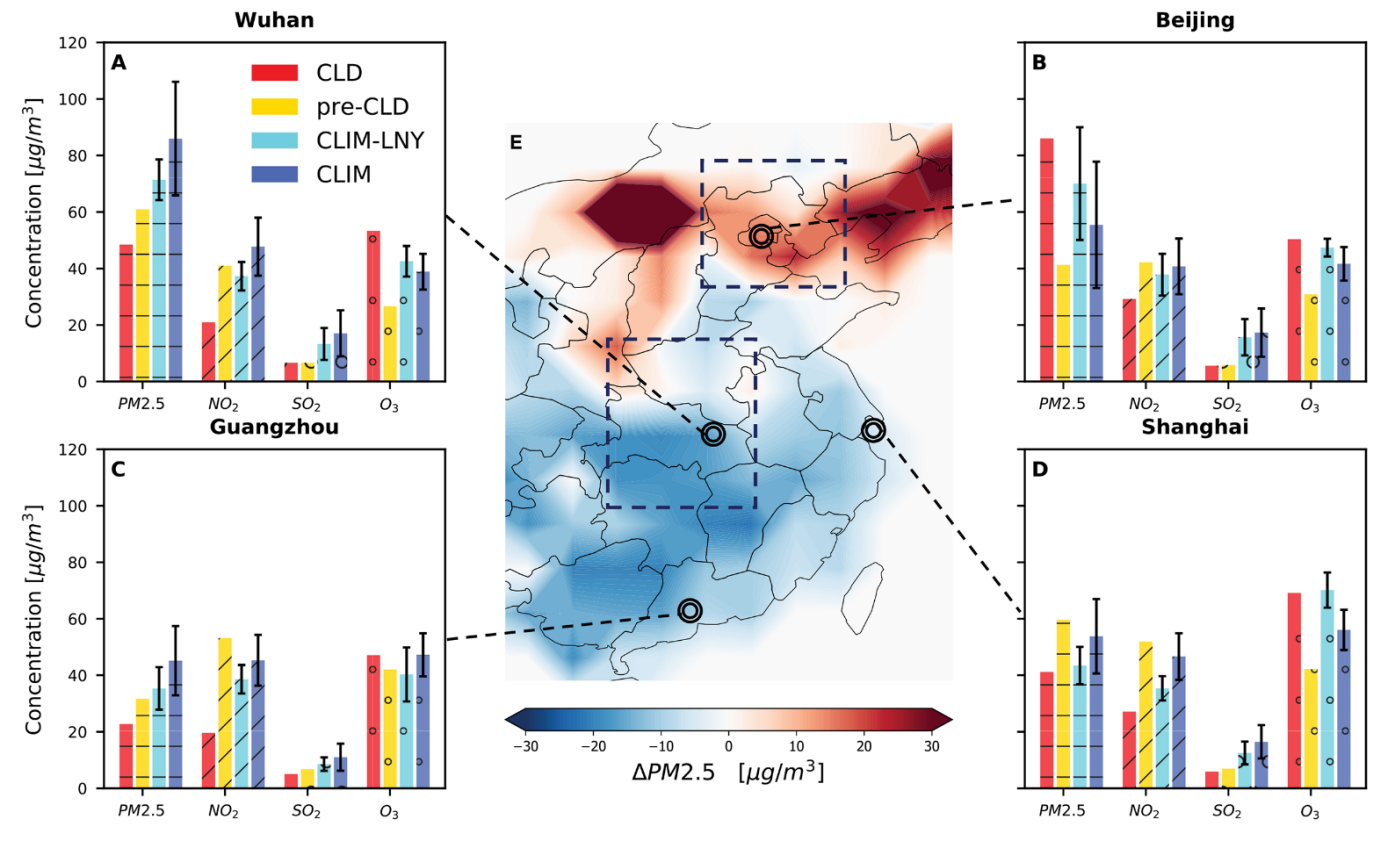
Ground-based station observation of particulate matter (aerodynamic diameter less than 2.5 μm, PM_2.5_), NO_2_, SO_2_, and ozone in eastern China including four megacities (A. Wuhan, B. Beijing, C. Guangzhou, and D. Shanghai). The figure compares the three-week averages during the city lockdown period (CLD), the three-week averages before the city lockdown (pre-CLD), the five-year climatology for 2015-2019 during the same period with CLD in the Chinese lunar calendar that covers the Lunar New Year (CLIM-LNY), and the five-year climatology for 2015-2019 during the same period with CLD in the Gregorian calendar (CLIM). Error bars indicate the standard deviations over multiple years. (**E**) The map of surface PM_2.5_ changes in 2020-CLD compared to CLIM-LNY based on the 1515 state monitoring stations (SI Fig. 2). The low-resolution patterns in the north and west are caused by the sparsity of stations. Two boxes indicate the Beijing-Tianjin-Hebei and central China regions. For ozone, 1 μg m^−3^ is approximately about 0.47 ppb under a standard condition.

In contrast to southern and central China, PM_2.5_ in northern China during the outbreak period experienced remarkable increases (Fig. 2B). During the three weeks of 2020-CLD, several severe haze events occurred in Beijing with the maximum daily PM_2.5_ level of 273.8 μg m^−3^. The CLD-mean surface PM_2.5_ in Beijing increased by 16.3 μg m^−3^ (+23.4%) and 30.6 μg m^−3^ (+55.1%) in comparison with CLIM-LNY and CLIM, respectively (Fig. 2B). Nonetheless, NO_2_ and SO_2_ remained the lowest among the past six years, similar to that of the southern cities. Response of ozone concentration in Beijing followed a similar trend as that of PM_2.5_, reaching a peak during the CLD. Daytime relationships between NO_2_ and ozone concentrations in the winter of northern China show remarkable ozone titration during daytime, particularly with increasing PM_2.5_ which further attenuates the incoming solar radiation, but the titration effect becomes considerably alleviated during the city lockdown (fig. S3). Nationwide, 1515 state monitoring stations show clear hot spots of surface PM_2.5_ over northern China during the 2020-CLD (Fig. 2E), although the national mean of the 2020-CLD PM_2.5_ was 52.1 μg m^−3^ which falls in the 1-σ range of variation of national climatology 54.7 ± 6.1 μg m^−3^. Satellite-observed aerosol optical depth (AOD) based on the Moderate Resolution Imaging Spectroradiometer (MODIS) corroborates the persistent haze over northern China. Significantly high levels of AOD (> 0.8) were present over the North China Plain but did not occur in any previous year since 2015 (fig. S4), leading to 40-100% increases in AOD during the city lockdown.

Possible factors that explain enhanced PM_2.5_ and ozone levels in the face of declining precursor gas emissions include the complex chemistry of secondary aerosols and ozone (*7*, *13*) as well as the meteorological influence (*8*). Changes in relative humidity (RH), near-surface wind speed and direction, planetary boundary layer (PBL) height, and precipitation between the 2020-CLD and CLIM-LNY are shown in Fig. 3, based on fifth-generation ECMWF global atmospheric reanalysis (ERA5). In northern China, which is climatologically dry during the wintertime, a larger than usual amount of moisture accumulated near the surface during the city lockdown, with a three-week mean RH of 55.2% and a maximum of 100%. Compared to the climatology, RH increased by 30-50% (Fig. 3A), facilitating multiphase reactions for aerosol formation and growth (*16*). Wind conditions were also favorable for haze formation; in Beijing, the mean wind speed decreased by 20%, and winds switched to southerly that normally originate from the polluted industrial regions in Hebei Province (Fig. 3B). Consistent with the increase in RH and the decrease in wind speed, PBL height in northern China generally declined during the city lockdown, inducing a stable boundary layer and stagnant air (Fig. 3C). As a result, both ozone and PM_2.5_ increased in Beijing. During the same period, as precipitation occurred mainly over southern China, no significant wash-out occurred in northern China, conducive for haze development during the city lockdown. Also note that, as positive feedback to the meteorological variations (*17*), aerosols can reduce PBL height and stabilize lower atmosphere via their radiative effects (*18*), and suppress light precipitation via their microphysical effects (*19*).

**Fig. 3:**
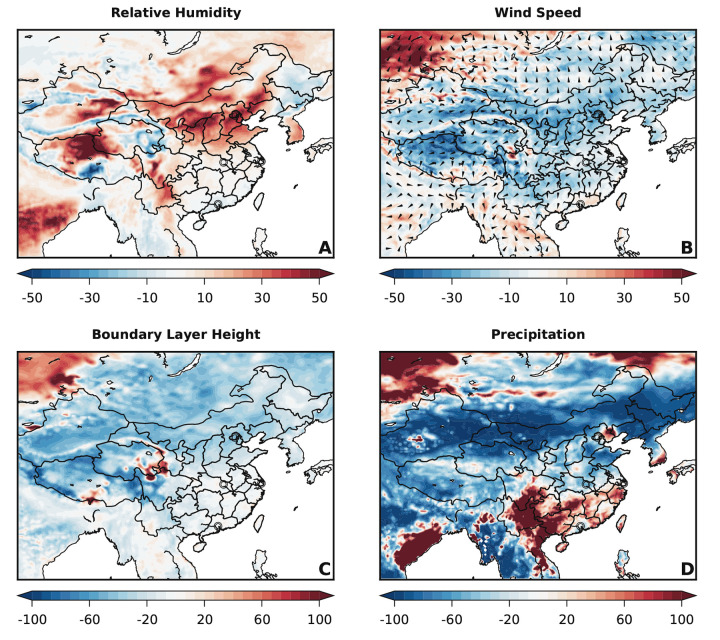
Fractional changes (%) in meteorological conditions between the 2020-CLD and the lunar new year climatology (CLIM-LNY) during 2015-2019 based on the ERA5 reanalysis data. (**A**) 1000-hPa relative humidity, (**B**) 10-m wind speed (contours) and wind direction (vectors), (**C**) boundary-layer height, and (**D**) daily precipitation. Symbols in the maps indicate the location of the four major cities in Fig. 2.

To reveal the physical and chemical mechanisms of the unexpected PM_2.5_ and ozone enhancement in northern China during the COVID19, we have conducted atmospheric chemistry and transport simulations using the Weather Research and Forecast model online coupled with full gaseous and aerosol chemistry (WRF-Chem). The unusual particulate levels during the 2020 CLD in the Beijing-Tianjin-Hebei area (BTH) are well reproduced in our baseline simulations, in terms of consistent peak values about 200 μg m^−3^, well simulated temporal evolution over the three weeks, and small mean bias (MB) about 2.6 μg m^−3^ (Fig. 4A). Surface ozone concentrations and diurnal cycles are comparable with ground-based observations (Fig. 4B), as with other precursor trace gases including SO_2_, NO_2_, and CO (fig. S5). Predicted aerosol chemical composition shows that organic aerosol (OA), nitrate, and sulfate are predominant species in BTH (fig. S6). When severe haze forms with a stable boundary layer and high humidity, inorganic fractions significantly increase with reduced OA, consistent with previous observations in the same area (*20*).

**Fig. 4:**
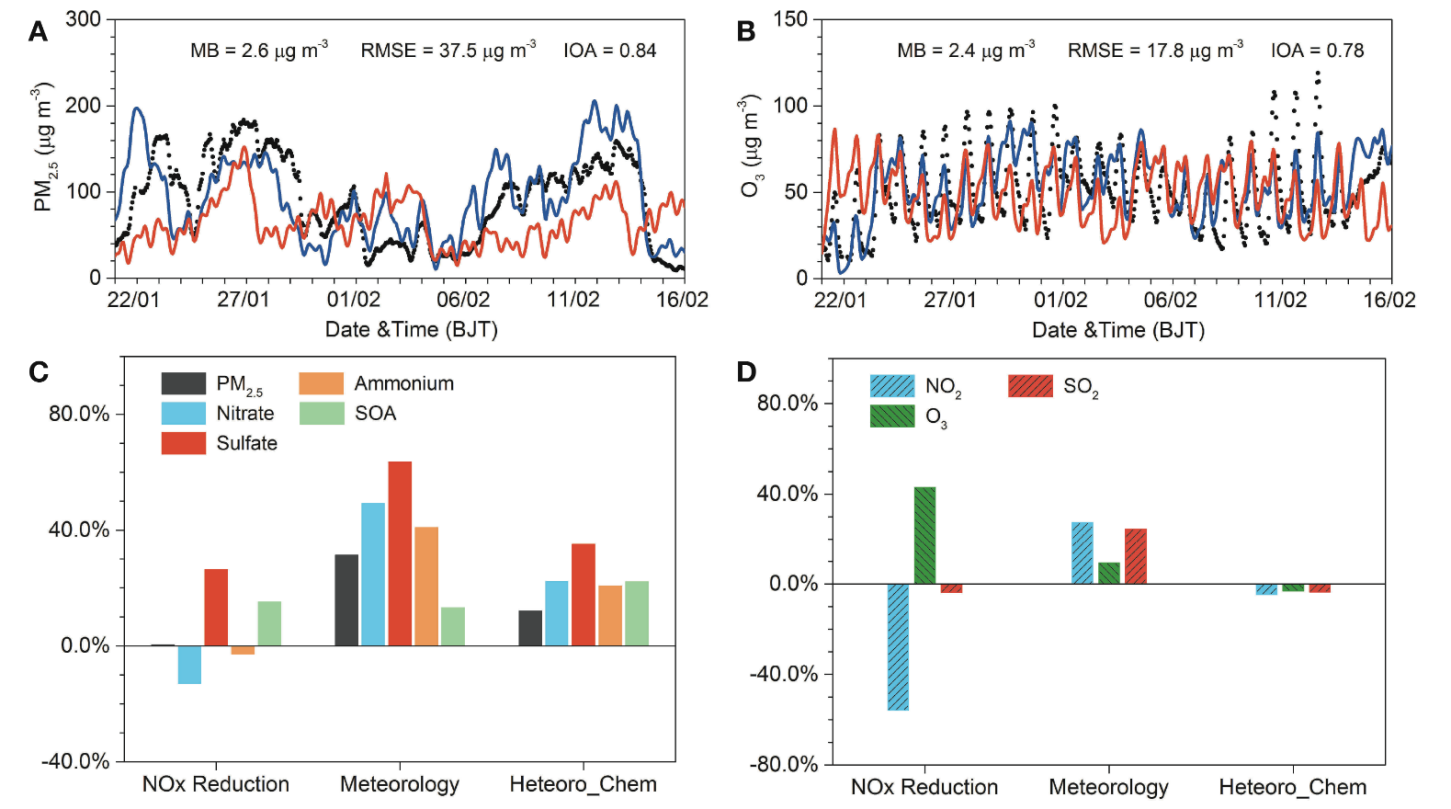
WRF-Chem simulated aerosol species and precursor gases during the COVID-19 city lockdown period in the Beijing-Tianjin-Hebei region, and their sensitivity to the altered emissions, meteorological conditions, and chemical pathways. (**A**) Time evolution of surface PM_2.5_ concentrations in the ground-based observations (black dots), the baseline simulation (blue line), and the sensitivity simulation with the climatological (2015-2019) meteorological conditions (red line, see details in table S3). (**B**) The same with (A) but for ozone. (**C**) The simulated fractional changes in different aerosol species in response to changes in NO_x_ emissions, meteorological conditions, and the representation of heterogeneous chemistry. (**D**) The same with (C) but for gaseous pollutants including NO_2_, SO_2_, and O_3_.

A series of model sensitivity simulations were conducted using altered emission rates, different meteorological conditions, and different sophistication of chemical schemes. An 80% NO_x_ emission reduction from all sectors in the model, consistent with the observed NO_2_ reduction during the city-lockdown period, induces a 13.0% reduction in nitrate aerosol but 26.3% and 15.1% increases in sulfate and secondary organic aerosol (SOA), respectively (Fig. 4C). The latter increases can be attributed to the enhanced atmospheric oxidizing capacity following the 42.9% ozone increase (Fig. 4D). Interestingly, the net PM_2.5_ change by NO_x_ reduction is not evident because of the cancellation of changes in different aerosol components. The meteorological influence on PM_2.5_ and ozone is assessed by comparing a pair of simulations with the meteorological conditions from this year and a multi-year climatology during the same period. It shows that due to the adverse ventilation conditions and anomalously high humidity during the city-lockdown period, all aerosol species are increased, with the largest fractional change of 63.5% for sulfate (Fig. 4C). Total PM_2.5_ is increased by 31.3% accordingly. Moreover, heterogeneous chemistry processes (see more details in METHOD) contribute positively to the aerosol formation and haze development during the city-lockdown period, due to the concurrent high humidity and aerosol water. Our model assessment shows a 12.0% increase in PM_2.5_ contributed from heterogeneous chemistry in northern China. Comparisons among the simulations altering emissions, chemistry, and meteorology reveal that the unprecedented NO_x_ reduction during the COVID 19 does not significantly reduce aerosol formation because of the non-linear ozone and aerosol chemistry. In addition, meteorological variations are crucial in the haze formation in northern China by trapping pollutants in the urban area and inducing more efficient aerosol formation from heterogeneous chemistry. Because high humidity and atmospheric stability were absent over central China, including Wuhan, a gradual decline of PM_2.5_ during the lockdown period can be seen in both ground-based observations and model simulations (fig. S7). An increasing trend of ozone can also be identified in the temporal evolution. Aerosol chemical compositions generally are maintained, with OA accounting for 36-40% of total aerosol mass, and sulfate-nitrate-ammonium for another 40% (fig. S6).

The COVID-19 outbreak led to unprecedented anthropogenic emission reductions from traffic and manufacturing sectors and the consequent city lockdowns. Hence, it offered a unique opportunity to assess the interplay between emissions, chemistry, and meteorology. Our synergistic analyses of the spatio-temporal distributions of PM and precursor gases, meteorological fields, and simulated PM formation pathways reveal a surprising PM exacerbation due to the unfavorable meteorological conditions, invigorated heterogeneous chemistry, and enhanced secondary aerosol formation with the elevated ozone oxidation capacity by NO_x_ reduction. In particular, our work provides unambiguous evidence that reduction in aerosol precursor emissions was compromised by multi-phase chemistry promoted by increased humidity. The role of multi-phase chemistry in haze formation is presently uncertain, and the findings here call for future research in this area.

Reductions in NO_x_ and SO_2_ from traffic and manufacturing sectors have long been considered as the normal protocol in implementing regulatory policies. Our work shows that such a protocol achieves only limited effects on PM and ozone levels, without simultaneous emission controls from power plants and heavy industry, such as petrochemical facilities. Therefore, we suggest a more comprehensive regulation of precursor gases from all possible sectors when developing an emission control strategy. For example, our model sensitivity experiments show 20% reduction in ozone and 5% reduction in PM_2.5_ by implementing 30% reductions of VOCs from all possible emission sources (fig. S8). As opposed to the previous “Olympic Blue” and “APEC Blue” shutdowns, an unexpected increase in PM levels in northern China occurred in a three-week period during the COVID-19 pandemic. The decisive role of meteorology in this unexpected haze formation in northern China during this episode underscores the importance of taking meteorological factors into account when short-term stringent emission controls are planned.

As the COVID-19 pandemic is still ongoing, the unexpected PM elevation has potentially profound implications for the airborne transmission of virus. A new emerging study shows plausible virus transmission via aerosols in populous areas (*21*). Meanwhile, an exposure to high levels of PM can cause adverse effects on the respiratory and cardiovascular systems and possibly increase the fatality rate of COVID-19 (*22*). Therefore, future work is urgently needed to establish the causal relationship between aerosol pollution and COVID-19.

## Supplementary Materials

science.sciencemag.org/cgi/content/full/science.abb7431/DC1 Materials and Methods Figs. S1 to S9 Tables S1 to S3 References (*23*–*41*)
